# Activation of IRE1 Endonuclease Activity Regulates Zika Virus Replication and Antiviral Response During Infection in Human Microglia

**DOI:** 10.3390/v17101291

**Published:** 2025-09-24

**Authors:** Tomás Hernández-Díaz, Aarón Oyarzún-Arrau, Aracelly Gaete-Argel, Delia López-Palma, Javier López-Schettini, Dominique Fernández, Fernando Valiente-Echeverría, Fabiola Osorio, Ricardo Soto-Rifo

**Affiliations:** 1 Laboratory of Molecular and Cellular Virology, Núcleo Interdisciplinario de Microbiología, Instituto de Ciencias Biomédicas (ICBM), Facultad de Medicina, Universidad de Chile, Independencia 1027, Santiago 8380453, Chile; tomas.hernandez@ug.uchile.cl (T.H.-D.); aaroneoa1@gmail.com (A.O.-A.); aracelly.gaete@ug.uchile.cl (A.G.-A.); delialopez@ug.uchile.cl (D.L.-P.); fvaliente@uchile.cl (F.V.-E.); 2 Millennium Institute on Immunology and Immunotherapy, Santiago 8331150, Chile; 3 Center for HIV/AIDS Integral Research, Faculty of Medicine, Universidad de Chile, Santiago 8380453, Chile; 4 Laboratory of Immunology and Cellular Stress, Núcleo Interdisciplinario de Farmacología e Inmunología, Instituto de Ciencias Biomédicas (ICBM), Facultad de Medicina, Universidad de Chile, Independencia 1027, Santiago 8380453, Chile; javi.apus@gmail.com (J.L.-S.); dominique.fernandez.q@gmail.com (D.F.); fabiola.osorio@uchile.cl (F.O.)

**Keywords:** Zika virus, microglia, ER stress, IRE1, innate immune response

## Abstract

Zika virus (ZIKV) can infect and replicate in the endoplasmic reticulum (ER) of different human cell types, including neural progenitor cells, radial glial cells, astrocytes, and microglia in the brain. ZIKV infection of microglia is expected to trigger both ER stress and the induction of an antiviral response through production of type-I interferons and pro-inflammatory cytokines, contributing to neuroinflammation during infection. Despite their critical role in ZIKV pathogenesis, the interplay between ER stress and the antiviral response during infection has not been fully characterized in human microglia. In this work, we show that infection of a human microglia cell line with ZIKV triggers the induction of an antiviral response and the activation of the endonuclease activity of the unfolded protein response sensor IRE1. Interestingly, we observed that both IRE1 and XBP1 were sequestered to the viral replication sites during infection. Moreover, pharmacological inhibition or hyperactivation of the endonuclease activity of IRE1 resulted in reduced viral titers. As such, while inhibition of IRE1 resulted in an increased type-I interferon response, hyperactivation led to a decrease in ZIKV RNA levels and the appearance of ER-derived cytoplasmic structures containing NS3, IRE1, and XBP1. Together, our data indicate that regulation of the endonuclease activity of IRE1 is critical for both ZIKV replication and immune activation, highlighting the potential of the ER stress sensor as a target for the development of antivirals to treat ZIKV infections.

## 1. Introduction

Zika virus (ZIKV) is a mosquito-borne virus classified by the World Health Organization as an important threat to the human population [1]. Its rapid spread across the Americas in 2015 and its association with both congenital Zika disease or Guillain-Barré syndrome raised major concerns regarding the pandemic potential of this virus and its impact on human health [2]. Unfortunately, the race for effective vaccines and specific antiviral treatments has been slowed by the complex interplay between ZIKV and the host immune system, revealing critical gaps in our understanding of the pathogenesis and immune evasion strategies employed by the virus [3,4]. ZIKV infects a wide range of human cells including placental cells, epithelial cells, and macrophages, as well as brain cells such as neural progenitor cells, radial glial cells, oligodendrocytes, astrocytes, and microglia [5,6,7,8,9,10,11]. Regardless of the cell type infected, the innate immune response is expected to mediate early detection and control of ZIKV replication. During infection, pattern recognition receptors (PRRs) recognize viral pathogen-associated molecular patterns (PAMP) triggering signaling cascades that culminate in the production of type I interferons (IFN-I) and pro-inflammatory cytokines [10,12,13,14]. On the other hand, ZIKV has evolved strategies to evade these host responses where non-structural proteins, such as NS1, NS4A, and NS4B, have been shown to interfere with IFN-I production by targeting various components of this pathway, dampening the establishment of an antiviral state [15,16,17].

ZIKV replication occurs in viral replication compartments (VRCs) assembled at the endoplasmic reticulum (ER), where key processes for viral replication, including viral protein and RNA synthesis as well as viral particle assembly, take place [18,19,20,21]. ZIKV replication triggers ER stress, which in turn induces a conserved cellular response known as the unfolded protein response (UPR) [22,23,24,25]. The UPR is an adaptive mechanism responsible for mitigating the cytotoxic effects of ER stress and restoring cellular homeostasis [26]. The UPR is regulated by three branches, each driven by an ER resident sensor: PKR-like ER kinase (PERK), activating transcription factor 6 (ATF6), and inositol-requiring enzyme 1 (IRE1) [27]. IRE1 is the most conserved UPR sensor, containing a kinase and an endoribonuclease domain, which processes the mRNA encoding XBP1s (X-box binding protein spliced), a transcriptional regulator of ER biogenesis-related genes. Also, upon acute ER stress conditions, IRE1 endoribonuclease can cleave additional mRNAs via a process termed ‘Regulated IRE1-Dependent Decay’ or RIDD, impacting inflammation and cell survival [26,27,28].

Recent evidence has provided links between IRE1 endonuclease activation as a direct regulator of IL-6 and TNF-α transcription through transcriptional activation mediated by XBP1s, enhancing their expression during conditions of ER stress [29,30]. The involvement of XBP1s in the regulation of cytokine production is further supported by their role in macrophage activation and the subsequent secretion of type-I IFN [31]. Additionally, IRE1 activation and XBP1 splicing also modulate the expression of a broader range of cytokines that include the production of IL-1β and chemokines, further highlighting their role in amplifying inflammatory responses [32]. Similarly, other studies have demonstrated that the IL-6 family of cytokines, including IL-11, are differentially regulated during ER stress in various cell types, such as astrocytes and macrophages, through mechanisms involving PERK and JAK1 signaling [33].

Understanding the complex interplay between ZIKV replication, ER stress, and the antiviral response is crucial to improving our knowledge of the virus–host interaction. In this work, we aimed to elucidate the dynamic interactions between ZIKV replication in the VRC assembled at the ER and the activation of the endonuclease activity of the ER stress sensor IRE1, together with the induction of the antiviral response in a human microglia cell line. Our data indicate that a tight regulation of the endonuclease activity of IRE1 is critical for both ZIKV replication and control of the antiviral response in this cellular model.

## 2. Materials and Methods

### 2.1. Cell Culture and Virus Infection

The human microglia C20 [34] and Vero E6 cell lines were cultured at 37 °C in a 5% CO_2_ atmosphere and maintained in DMEM culture media (Gibco, 12800-017, Waltham, MA, USA) supplemented with Antibiotic-Antimycotic 1% (Sigma-Aldrich, A5955, Darmstadt, Germany), L-glutamine 1% (Gibco, A29168-01, Waltham, MA, USA), and 10% FBS (Sigma-Aldrich, F0926, Darmstadt, Germany). For Zika virus infection, 3.5 × 10^5^ C20 cells were seeded in 60 mm plates, and the virus diluted in DMEM 2% was adsorbed at a MOI 3 for 1 h at 37 °C in a 5% CO_2_ atmosphere. After adsorption, the inoculum was removed, and cells were washed with sterile PBS 1X and incubated with culture media for the indicated times.

### 2.2. Viral Production and Propagation

ZIKV isolate BeH819015 (GI: 975885966) was obtained from the pCC1-SP6-ZIKV vector previously described [35]. Briefly, the pCC1-SP6-ZIKV vector (kindly donated by Dr. Andres Merits) was used as a template for in vitro transcription using the mMessage mMachine SP6 kit (ThermoFisher Scientific, AM1340, Waltham, MA, USA) to produce infective RNA (iRNA). Vero E6 cells were transfected with 5 µg of iRNA using Lipofectamine™ MessengerMAX™ Transfection Reagent (ThermoFisher Scientific, #LMRNA015, Waltham, MA, USA) and after 3 days, infectious ZIKV was recovered from the supernatant and virus stock was stored at −80 °C in 50% FBS until use. Viral titers from viral stocks and supernatants of infected microglia were determined by the plaque lysis assay in Vero E6 cells as previously described [36].

### 2.3. PCR and RT-qPCR

Total RNA was extracted with the TRIzol™ Reagent (Invitrogen, 15596026, Waltham, MA, USA), and cDNA was synthesized using the High-Capacity cDNA™ Reverse Transcription Kit (Applied Biosystems, 4368814, Waltham, MA, USA), following the manufacturer’s instructions. Real-time PCR was performed using the Brilliant II SYBR^®^ Green QPCR Master Mix (Agilent, 600828, Santa Clara, CA, USA), following the manufacturer’s instructions, in an AriaMx Real-Time PCR System (Agilent, Santa Clara, CA, USA). The data were analyzed by the 2^−ΔΔCT^ method using 18S rRNA as a housekeeping gene. Conventional PCR was performed using the GoTaq^®^ Green Master Mix (Promega Corporation, Madison, WI, USA) to evaluate XBP1 mRNA splicing. The PCR amplicons were resolved on a 3% agarose gel stained with SafeView Plus (Fermelo Biotec, Santiago, Chile) and analyzed using the Alliance Q9 Advanced system (UVITEC) and FIJI/ImageJ software v1.54p (NIH, USA). Table 1 shows the sequences of primers used in this study.

### 2.4. Western Blot Analysis

Cells were lysed in RIPA buffer, and protein concentration was determined using the bicinchoninic acid assay (ThermoScientific, 23227, Waltham, MA, USA). Samples were boiled at 95 °C for 10 min, and 30 μg of total protein was resolved by SDS-PAGE and transferred to polyvinylidene fluoride (PVDF) membranes (Millipore, Burlington, MA, USA). Then, membranes were blocked and incubated with primary antibodies overnight at 4 °C. Primary antibodies used were anti-GRP78/BiP (Cell Signaling Technology, C50B12, Danvers, MA, USA); anti-β-actin (Santa Cruz Biotechnologies, sc-47778, CA, USA); and anti-NS3 (kindly provided by Dr. Andres Merits). Secondary horseradish peroxidase-conjugated antibodies were obtained from Jackson Immunoresearch (Baltimore, PA, USA). Membranes were analyzed with the ECL Western blotting substrate (Pierce, 32106, Waltham, MA, USA) and images were captured and quantified using the Alliance Q9 Advanced system (UVITEC, Cambridge, UK).

### 2.5. Flow Cytometry and Cytometric Bead Array

Flow cytometry was performed on a BD LSR Fortessa (BD Biosciences, San Jose, CA, USA) flow cytometer using FACSDiva software (BD Biosciences, San Jose, CA, USA). Cytometric bead array (CBA) was performed using the BD^™^ Cytometric bead array Human Inflammatory Cytokine kit (BD Biosciences, San Jose, CA, USA). To this end, culture supernatant was collected, and cytokines were quantified following manufacturer’s instructions. Flow cytometry data was analyzed using FlowJo software v10 (FlowJo, LLC, Ashland, OR, USA).

### 2.6. Pharmacological Treatment

C20 human microglia cells were seeded at 3.5 × 10^5^ cells per well in 6-well culture plates for each assay and were maintained at 37 °C and 5% CO_2_. After seeding, cells at 80% confluence were treated with DMSO (control) or Tunicamycin (Tn) at 1 µg/mL for 24 h [37]. For assays involving activation and inhibition of IRE1, the IRE1 endonuclease activity agonist IXA4 [38] and the IRE1 endonuclease activity inhibitor STF-083010 (STF) [39] were used and both compounds were diluted in DMSO. Cells were treated with DMSO (control), IXA4 at 10 µM or STF-083010 at 60 µM for one hour prior to viral inoculation. Viral adsorption and assays were always conducted in the presence of the corresponding drugs or DMSO as control.

### 2.7. Indirect Immunofluorescence

Human microglia cells were seeded on 12 mm coverslips and were treated with STF-083010 or IXA4 and then infected for 24 h. After that time, supernatant was removed, cells were fixed with consecutive incubations and washes with methanol-free PFA at 4% (Pierce, 28906, Waltham, MA, USA) for 20 min, glycine 0.1 M for 10 min, and finally, permeabilized with TritonX-100 0.2% for 5 min. For indirect immunofluorescence protocol, cells were incubated in blocking solution diluted in PBS 1X (Roche, 11585762001, Basel, Switzerland) for 30 min at room temperature and then incubated with primary antibodies at 37 °C in a wet chamber for 1 h. Primary antibodies used were anti-IRE1α (Santa Cruz Biotechnologies, sc-390960, CA, USA), anti-XBP1 (amino acids 76-263 from the C-terminus of XBP-1, Santa Cruz Biotechnologies, sc-8015, CA, USA), and anti-NS3 (kindly provided by Dr. Andres Merits). Then, cells were washed three times with PBS 1X and incubated with Alexa Fluor conjugated secondary antibodies (Invitrogen, Waltham, MA, USA) at 37 °C in a wet chamber for 1 h. After secondary antibody incubation, coverslips were washed three times with PBS 1X and incubated with DAPI solution (Invitrogen, Waltham, MA, USA) for 5 min at room temperature. Finally, coverslips were mounted, and images were acquired with an Olympus IX73 inverted microscope (Olympus America Inc., Center Valley, PA, USA) using the 60X objective. Confocal images were acquired using a Zeiss LSM 700 Zeiss inverted microscope (Zeiss, Germany) and analyzed using the FIJI/ImageJ software v1.54p (NIH, USA).

### 2.8. RNA Fluorescence In Situ Hybridization

To perform RNA-fluorescent in situ hybridization (RNA-FISH), C20 microglia cells were seeded on coverslips. After infection and treatment with DMSO and IXA4, cells were fixed with 4% paraformaldehyde for 20 min, incubated with 0.1 M glycine for 10 min and permeabilized with 0.2% Triton X-100 in 1X PBS for 5 min at room temperature. Fifty nanograms of digoxigenin-labeled ZIKV RNA probe per coverslip were hybridized overnight at 37 °C in a solution containing 2 mM Vanadyl Ribonucleoside Complex, 0.02% BSA, 50% formamide, and 1.5 µg/µL tRNA. Coverslips were then washed with a 50% formamide-0.2X Saline-Sodium Citrate (SSC) solution for 30 min at 50 °C and then incubated with primary antibodies in antibody solution (2X SSC solution, 8% formamide, 2 mM Vanadyl Ribonucleoside Complex, 0.02% BSA) for 1 h at 37 °C. The primary antibodies used were the following: anti-digoxigenin (sheep 1:200, Roche, Basel, Switzerland) and anti-NS3 (rabbit 1:100, kindly donated by Dr. Andres Merits). After incubation, unbound primary antibodies were washed 3 times with 1X PBS and incubated with the respective Alexa Fluor conjugated secondary antibodies (1:500, Invitrogen, Waltham, MA, USA). Finally, coverslips were stained with DAPI (Invitrogen, Waltham, MA, USA), washed, and mounted on slides using aqueous mounting solution (Sigma-Aldrich, Darmstadt, Germany). Confocal images were obtained with a Zeiss LSM 700 Confocal Microscope (Zeiss, Germany) and processed using FIJI/ImageJ software v1.54p (NIH, USA).

### 2.9. Statistical Analysis

Data are presented as mean ± SEM of three independent experiments. An unpaired, two-tailed Student’s *t*-test was used to compare two groups at a time, and for comparisons of more than two groups, one-way ANOVA was used. Analysis was conducted in GraphPad Prism v9.0 (Boston, MA, USA). *p*-value < 0.05 was considered statistically significant and *p* values are indicated in plots.

## 3. Results

### 3.1. ZIKV Infection Triggers ER Stress and a Pro-Inflammatory Response in Human Microglia

**Figure 1:**
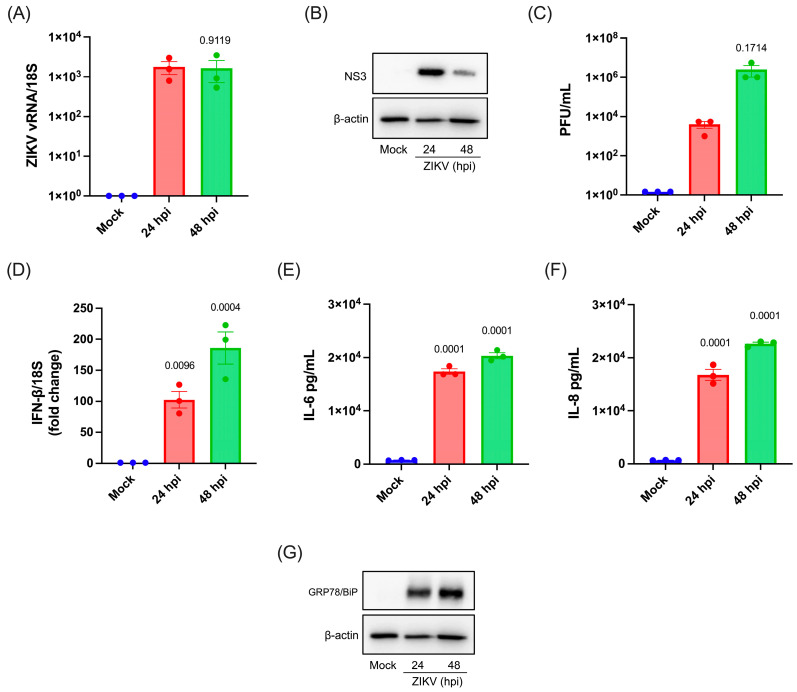
ZIKV infection on human microglia cells and the innate immune response. (**A**) Intracellular ZIKV vRNA quantification by RT-qPCR from human microglia at 24 and 48 hpi. (**B**) Western blot analysis of ZIKV-infected human microglia at 24 and 48 hpi showing the viral protease NS3 and β-actin as loading control. The image is representative of three independent experiments. (**C**) Plaque-forming units (PFU) from cell culture supernatants obtained from ZIKV-infected human microglia at 24 and 48 hpi. (**D**) Relative expression of IFN-β mRNA of human microglia infected with ZIKV at MOI 3 for 24 and 48 hpi. (**E**) Cytometric bead array for the quantification of IL-6 released to the supernatants of human microglia infected at 24 and 48 hpi. (**F**) Cytometric bead array for quantification of IL-8 released to supernatants of human microglia infected at 24 and 48 hpi. (**G**) Western blot showing the cellular protein GRP78/BiP as a marker of ER stress and UPR induction and β-actin as a loading control. The image is representative of three independent experiments. All plots are represented as mean ± SEM of three independent experiments. Statistical analysis was performed to compare the mock (or 24 hpi) condition using one-way ANOVA. A *p*-value of <0.05 was considered significant compared to 24 hpi in (**A**,**C**) or mock condition in (**D**–**F**), and *p*-values are indicated in plots.

Since microglia represent an important target cell during ZIKV infection contributing to neuroinflammation and neuronal injury in the brain [40,41], we sought to characterize viral replication using the adult human microglia cell line C20. For this, we infected human microglia with ZIKV and followed replication by measuring the intracellular levels of the viral protease/helicase NS3 and viral RNA (vRNA) as well as viral titers in supernatants at 24 and 48 h post-infection (hpi). We detected the intracellular accumulation of the vRNA reaching a peak at 24 hpi that was maintained at 48 hpi (Figure 1A). We also observed the expression of the viral RNA helicase NS3 at 24 and 48 hpi (Figure 1B). In addition, the production of infectious viral particles was detected at 24 hpi and 48 hpi (Figure 1C), indicating that a complete replication cycle occurs during the first 24 h of infection. Furthermore, a marked induction of the transcript-coding IFN-β was noticed at 24 and 48 hpi, indicating sustained activation of the type-I IFN response triggered by ZIKV replication (Figure 1D). We also detected a strong increase in IL-6 and IL-8 levels in the supernatants of infected microglia, indicative of the activation of a pro-inflammatory cytokine response during viral replication (Figure 1E,F). Finally, we observed an increase in the levels of the ER chaperone GRP78/BiP in infected microglia, indicating that ZIKV replication triggered canonical ER stress (Figure 1G).

Together, these results indicate that ZIKV infects and replicates in human microglia, triggering ER stress and the activation of an antiviral response through type-I IFN and pro-inflammatory cytokine responses.

### 3.2. ZIKV Infection Induces Activation of the IRE1 Endonuclease Domain in Human Microglia

**Figure 2:**
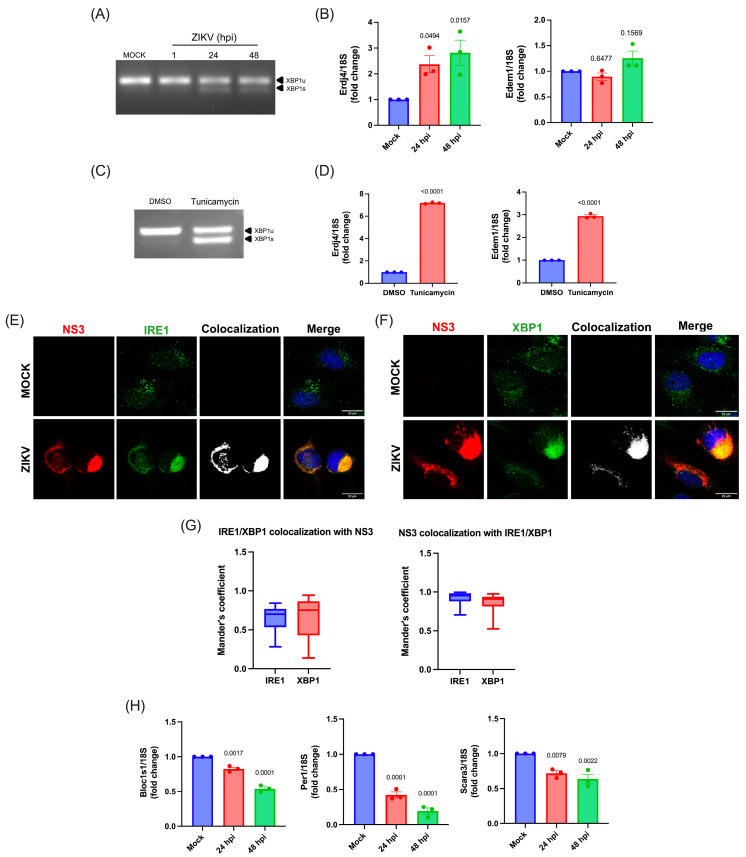
Effect of ZIKV infection on the IRE1/XBP1 axis activation. (**A**) Agarose gel of conventional RT-PCR products of the XBP1 mRNA during ZIKV infection at 1, 24 and 48 hrs. Unspliced (u) and spliced (s) forms of the XBP1 mRNA are indicated. (**B**) Relative expression of XBP1s targets, Erdj4 and Edem1, from human microglia at 24 hpi. (**C**) Agarose gel of conventional RT-PCR products of the XBP1 mRNA during tunicamycin treatment of human microglia cells. Unspliced (u) and spliced (s) forms of the XBP1 mRNA are indicated. DMSO was used as control vehicle. (**D**) Relative mRNA expression of XBP1s targets, Erdj4 and Edem1, from human microglia treated with tunicamycin (1 µg/mL) for 24 h. (**E**) Human microglia cells were infected with ZIKV for 24 h. Confocal images of microglia cells incubated with anti-IRE1 in green and anti-ZIKV NS3 in red. Nuclei were stained with DAPI. Images are representative of three independent experiments. Scale bar = 20 µm. (**F**) Human microglia cells were infected with ZIKV for 24 h. Confocal images of microglia cells were incubated with anti-XBP1 in green and anti-ZIKV NS3 in red. Nuclei were stained with DAPI. Images are representative of three independent experiments. Scale bar = 20 µm. (**G**) Mander’s coefficients showing the fraction of IRE1 or XBP1 signal overlapping with NS3 (**left panel**) and NS3 with IRE1 or XBP1 (**right panel**) (N = 21 cells for IRE1/NS3 and 17 cells for XBP1/NS3). (**H**) Relative mRNA expression of RIDD targets, Block1s, Scara3, and Per1 from human microglia at 24 hpi. All data are represented as mean ± SEM of three independent experiments. Statistical analysis was performed to compare the mock or DMSO condition using one-way ANOVA. A *p*-value of <0.05 was considered significant and *p*-value are indicated in plots.

Considering the induction of ER stress during infection together with the critical role of IRE1 in ZIKV replication in human cell models such as HeLa and A549 cells [42,43], we evaluated the activation of IRE1α (hereafter referred as IRE1) endonuclease activity during ZIKV infection at 24 and 48 hpi in the C20 human microglia cell line. We observed the non-canonical splicing of the XBP1 mRNA (XBP1s) in ZIKV-infected microglia at 24 and 48 hpi indicating the activation of the IRE1 endonuclease activity upon infection (Figure 2A). However, analyses of the XBP1s targets, Erdj4 and Edem1, by RT-qPCR showed that only the former was induced, suggesting a partial activation of the IRE1/XBP1 axis in infected cells (Figure 2B). Consistent with this idea, induction of ER stress and the IRE1/XBP1 axis by treatment with tunicamycin (Tn) revealed both a potent splicing of the XBP1 mRNA and the induction of Erdj4 and Edem1 transcripts; thus, confirming a partial activation of XBP1s during ZIKV infection (Figure 2C,D).

Interestingly, immunostaining and confocal microscopy analyses revealed that IRE1 and XBP1 colocalized together with the viral protein NS3 at the viral replication sites, suggesting that XBP1s might not reach the nucleus to activate its target genes (Figure 2E,F). Calculations of Mander’s coefficients suggest that while most of the NS3 signal colocalizes with IRE1 and XBP1, there are still some signals of both cellular proteins that are not recruited to the replication sites together with NS3 (Figure 2G).

To determine activation of the IRE1 endonuclease activity through the RIDD branch during ZIKV infection in human microglia, we measured expression of canonical RIDD targets Bloc1s1, Scara3, and Per1. Interestingly, our results revealed a significant decrease in the levels of these three transcripts, particularly noticed at 48 hpi. These data indicate that ZIKV infection induces activation of RIDD in the human microglia cell line (Figure 2G). Together, these results indicate that ZIKV infection activates the endonuclease activity of IRE1, triggering both the IRE1/XBP1s and IRE1/RIDD pathways in human microglia. However, the IRE1/XBP1s axis might be interfered by XBP1 retention at the viral replication sites.

### 3.3. IRE1 Endonuclease Activity Regulates ZIKV Replication in Human Microglia

**Figure 3:**
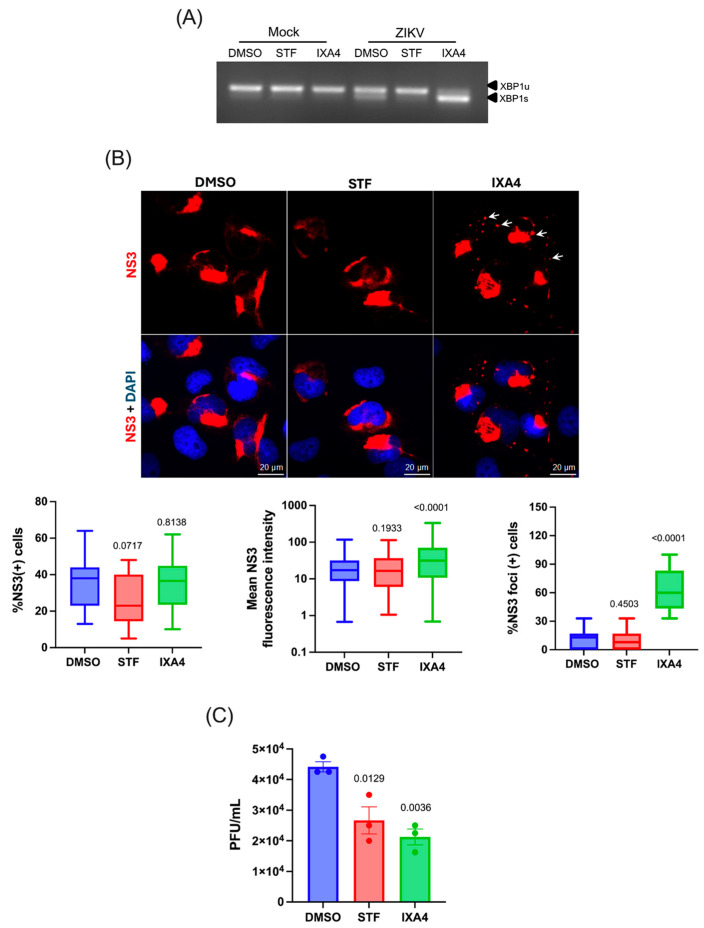
Effect of IRE1 endonuclease activity on ZIKV replication in infected human microglia. (**A**) Conventional RT-PCR analyses for the splicing of XBP1 mRNA, showing the unspliced (XBP1u) and the spliced form (XBP1s) in mock and ZIKV-infected microglia at 24 hpi treated with DMSO, STF-083010 (60 µM), or IXA4 (10 µM). (**B**) Human microglia cells were treated with STF-083010 (60 µM), or IXA4 (10 µM) and infected with ZIKV (MOI 3) for 24 h. Percentage of ZIKV NS3-positive cells in ZIKV-infected microglia (**left graph**), NS3 mean fluorescence intensity calculated from epifluorescence images (**middle graph**), and percentage of ZIKV NS3 foci positive cells (**right graph**) in ZIKV-infected microglia treated with DMSO (N = 169 cells), STF (N = 187 cells), IXA4 (N = 172 cells) are shown. White arrows indicate NS3-containing cytoplasmic foci in IXA4-treated cells. (**C**) Viral titers from supernatants from ZIKV-infected microglia treated with DMSO, STF-083010, or IXA4. All data are represented as mean ± SEM of three independent experiments. Statistical analysis was performed to compare DMSO condition using one-way ANOVA. A *p*-value of <0.05 was considered significant, and *p*-values are indicated in plots.

Given that the data presented here showed IRE1 accumulation at the viral replication sites, we then sought to evaluate whether activation of IRE1 endonuclease activity was involved in viral replication. For this, we took advantage of STF-083010 (STF), a pharmacological inhibitor of the IRE1 endonuclease activity and IXA4, a recently reported activator of the IRE1/XBP1s axis [38]. As expected, treatment with STF inhibited the splicing of XBP1 mRNA induced by ZIKV infection while treatment with IXA4 boosted XBP1 splicing in ZIKV infected cells (Figure 3A). Then, to investigate the impact that the modulation of the IRE1 endonuclease activity has on viral replication, we analyzed the effects of STF and IXA4 on the levels and subcellular localization of NS3 by epifluorescence microscopy. We observed that pharmacological inhibition or activation of the IRE1 endonuclease activity had no effect on the number of NS3 positive cells, suggesting that the UPR sensor might not be involved in the early steps of viral replication (Figure 3B, see left graph). However, while inhibition of IRE1 activity with STF had no effect on viral protein accumulation as judged by the mean fluorescence intensity (MFI) quantification of the NS3 signal, IRE1 activation by IXA4 treatment resulted in a significant increase in the MFI associated with the viral protein (Figure 3B, see middle graph). Interestingly, we also observed that treatment with IXA4 but not STF resulted in a dramatic change in the morphology of the viral replication sites, which also included the appearance of NS3-containing cytoplasmic foci (Figure 3B, see white arrows and right graph).

To further investigate the impact of IRE1 endonuclease activity on viral replication, we determined the effects of STF and IXA4 on the production of infective virions. Interestingly, we observed a significant reduction in viral titers in cells treated with both STF and IXA4, suggesting that optimal IRE1 endonuclease activity is required for the release of infectious virions (Figure 3C). Altogether, these results suggest that tight regulation of IRE1 endonuclease activity is critical for efficient ZIKV replication in human microglia.

### 3.4. Hyperactivation of the IRE1 Endonuclease Activity Induces the Appereance of Cytoplasmic Structures Derived from the ER

**Figure 4:**
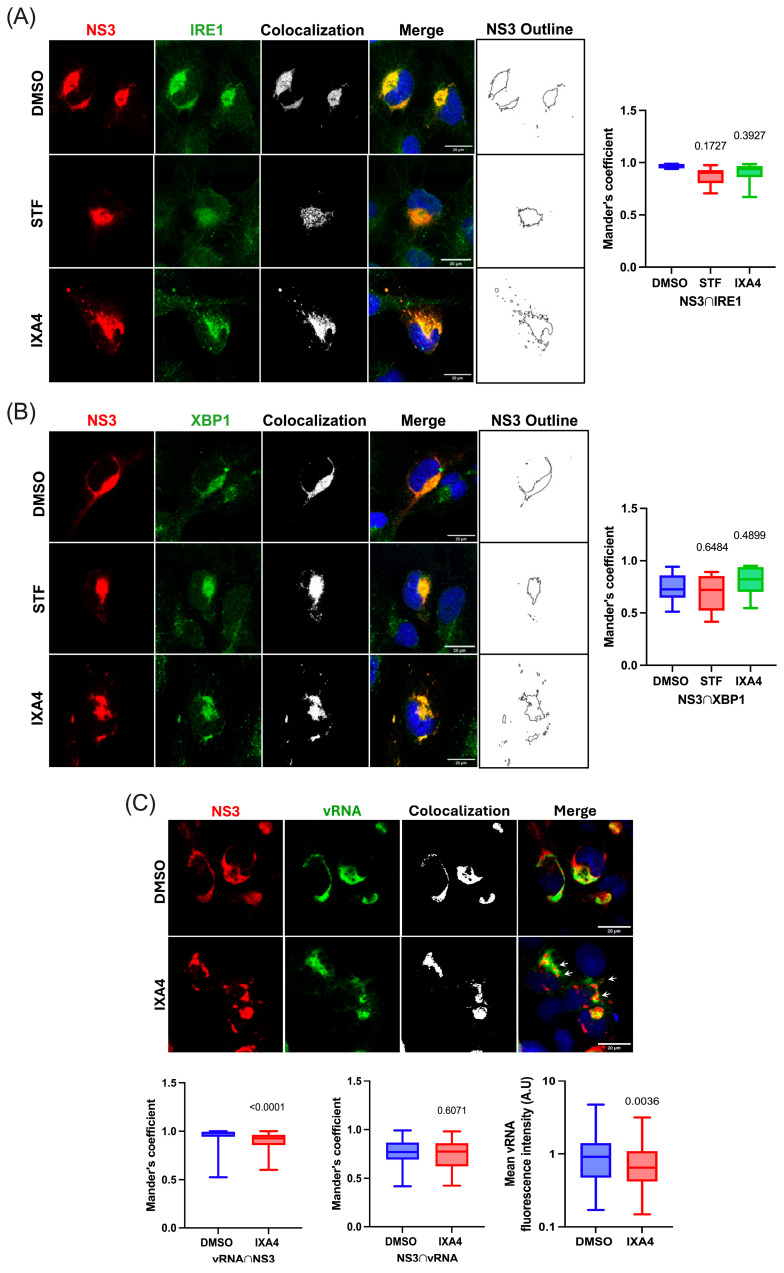
Effect of hyperactivation of the IRE1 endonuclease activity on ZIKV replication. (**A**,**B**) Confocal microscopy images of microglia cells treated with STF and IXA4 and incubated with anti-ZIKV NS3 together with anti-IRE1 (**A**) or anti-XBP1 (**B**) antibodies. Nuclei were stained with DAPI. The outline map of NS3 is shown to illustrate the morphological changes in the ER and the appearance of cytoplasmic structures in cells treated with IXA4. Images are representative of three independent experiments. Scale bar = 20 µm. Mander’s coefficients showing the co-occurrence of NS3 with IRE1 ((**A**), N = 19 cells) or XBP1 ((**B**), N = 31 cells). (**C**) Confocal microscopy images of RNA-FISH experiments of ZIKV-infected microglia treated with DMSO or IXA4. ZIKV vRNA was detected with a digoxigenin-labeled probe and anti-digoxin antibody, and NS3 was detected with anti-ZIKV NS3 antibody. Nuclei were stained with DAPI. White arrows indicate NS3-containing cytoplasmic foci in IXA4-treated cells. Images are representative of three independent experiments. Scale bar = 20 µm. Mander’s coefficient for the fraction of the vRNA signal overlapping with NS3 signals (**left graph**) and NS3 versus vRNA (**middle panel**) in cells treated with DMSO (N = 93 cells) or IXA4 (N = 93 cells). The MFI of vRNA signal in cells treated with DMSO or IXA4 is also shown (**right graph**). All data are represented as mean ± SEM of three independent experiments. Statistical analysis was performed to compare DMSO condition using one-way ANOVA. A *p*-value of <0.05 was considered significant and *p*-values are indicated in plots.

We were intrigued by the appearance of NS3-containing cytoplasmic foci in ZIKV-infected cells treated with IXA4 and thus, decided to further investigate this phenomenon. For this, we conducted confocal microscopy analyses in ZIKV-infected microglia treated with DMSO (as a control), STF, and IXA4 and looked to see whether IRE1 and XBP1 colocalized with NS3 in these cytoplasmic structures. These analyses confirmed that IRE1 and XBP1 colocalize together with NS3 at the replication sites in control cells and in cells treated with STF (Figure 4A,B, compares DMSO and STF). We also observed that both IRE1 and XBP1 colocalize with NS3 in cells treated with IXA4 (Figure 4A,B, see IXA4). From the images analyzed, we also confirmed that treatment with IXA4 resulted in evident morphological changes in the replication sites including the formation of cytoplasmic structures (Figure 4A,B, see the NS3 outline map). Interestingly, we observed that both IRE1 and XBP1 were delocalized together with NS3 at these cytoplasmic structures, indicating that they might be derived from the ER (Figure 4A,B, see colocalization points). Of note, Mander’s coefficient calculations revealed that the colocalization of NS3 together with IRE1 or XBP1 was unchanged by treatments with STF and IXA4, further suggesting that these proteins are delocalized together in IXA4-treated cells (Figure 4A,B, right graphs). We then investigated whether these ER-derived structures induced by the hyperactivation of IRE1 endonuclease activity corresponded to VRC that were split out from the ER. Since vRNA is a major component of the VRC, we looked at the localization of the vRNA in cells treated with DMSO (as a control) and IXA4 by RNA-FISH and confocal microscopy. We observed that while most of the vRNA signal colocalizes with NS3 at the replication sites, not all the NS3 signal colocalized with vRNA, indicating that NS3 also localizes at sites within the ER that are distinct from the VRC (Figure 4C, see DMSO and Mander’s coefficients). Interestingly, we observed that vRNA was not delocalized to the ER-derived cytoplasmic structures together with NS3, indicating that these cytoplasmic foci do not correspond to VRCs that are split out from the ER (Figure 4C, see white arrows in IXA4 and left-middle graphs). Of note, quantification of the MFI from the vRNA signal suggests that IXA4 treatment results in decreased vRNA levels (Figure 4C, see right graph). Altogether, these results strongly suggest that hyperactivation of the IRE1 endonuclease activity results in both decreased vRNA levels and the split of ER-derived structures containing NS3, IRE1, and XBP1, but not vRNA, which might account for reduced viral titers.

### 3.5. The IRE1 Endonuclease Activity Regulates the Antiviral Response Triggered by ZIKV in Human Microglia

**Figure 5:**
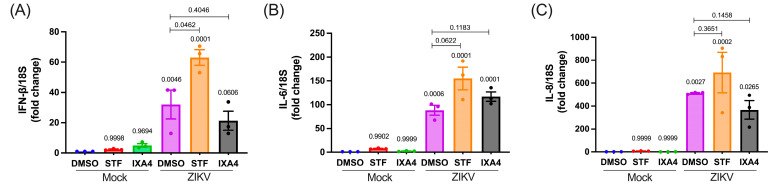
Effect of IRE1 activity modulation on innate immune response in infected human microglia. Relative expressions of (**A**) IFN-β, (**B**) IL-6 and (**C**) IL-8 transcripts in mock and ZIKV-infected microglia at 24 hpi, treated with DMSO, STF-083010 (60 µM), or IXA4 (10 µM). All data are represented as mean ± SEM. Statistical analysis using an ANOVA test was performed to compare the DMSO mock condition. N = 3. For the DMSO and STF/IXA4 comparison in infected conditions, a *t*-test against a DMSO control was conducted. A *p*-value of <0.05 was considered significant, and *p*-values are indicated in plots.

Although the reduction in viral titers observed in cells treated with IXA4 could be explained by reduced levels of the vRNA together with the deformation of the ER and the delocalization of the protease/helicase NS3 from the replication sites, the similar effect observed in cells treated with STF could not be explained by our immunofluorescence and confocal microscopy analyses. Considering the increasing number of reports linking the IRE1/XBP1s axis with the activation of innate immunity in different biological contexts including ZIKV infection [23,44,45,46], together with our data showing that the virus induces an antiviral response in human microglia, we aimed to determine whether inhibition or activation of IRE1 endonuclease activity affects the antiviral response. To this end, we analyzed expressions of IFN-β, IL-6, and IL-8 transcripts in mock and ZIKV-infected microglia treated with STF and IXA4, or DMSO as a control. In agreement with data from Figure 1, we observed that ZIKV infection resulted in increased expression of the mRNA coding for IFN-β, IL-6 and IL-8 (Figure 5A–C). Interestingly, inhibition of IRE1 endonuclease activity with STF resulted in significantly increased levels of IFN-β mRNA by infection, which was not observed under treatment with IXA4 (Figure 5A). Such an effect was not observed for the transcripts coding IL-6 and IL-8 suggesting a specific regulation of the IFN-β mRNA (Figure 5B,C). Together, these results suggest that inhibition of IRE1 endonuclease activity may result in a more potent induction of the type-I interferon response resulting in decreased viral titers.

## 4. Discussion

Zika virus is considered a pathogen of high risk according to the recent Pathogens Prioritization R&D Blueprint from the WHO [1]. Indeed, given its ability to reach the brain and cause neurological complications in newborns, infants, and adults, ZIKV represents a major threat for human health. Since microglia are the sentinels of the brain but also represent an important target for ZIKV in the central nervous system [9,47,48,49], the data presented here provide new and valuable information on this virus–cell-type interaction, contributing to improving our understanding of the pathogenesis associated with microglia infection. Our results obtained in an adult microglia cell line revealed active viral replication and the release of infective viral particles despite a significant induction of the IFN-β mRNA levels, as well as increased secretion of pro-inflammatory cytokines IL-6 and IL-8. Our observations are consistent with previous studies by Oliveira and colleagues, which also observed a pro-inflammatory response during ZIKV infection in the mouse microglia cell line BV-2 [50]. Interestingly, studies using primary neurons and microglia from immunocompetent mice aimed at understanding the neurological disorders and cognitive defects caused by ZIKV infection in adults revealed that mostly neurons and not microglia were infected [51], results that are probably influenced by the inability of ZIKV to counteract mouse STAT2 [52]. More recently, studies using human fetal brain explants proposed astrocytes as the main target cells for ZIKV being responsible for the production of a type-I IFN response [10]. Unfortunately, these fetal brain explants contained very low levels of microglia and thus, the contribution of these cells to viral replication and immune activation was not evaluated [10]. Indeed, Lum and colleagues reported that microglia are the main targets for ZIKV infection, being the major contributors to neuroinflammation during congenital ZIKV pathogenesis [40]. These last observations highlight the relevance of performing studies in human microglia models in which viral replication occurs in the presence of an antiviral and pro-inflammatory environment that the virus must counteract to successfully replicate and spread the infection.

Consistent with previous reports showing the ability of ZIKV to induce ER stress [23,24,25], we provide evidence for a marked increase in the ER chaperone GRP78/BiP during ZIKV infection, indicative of ER stress in infected microglia. We also demonstrate the activation of the ER stress sensor IRE1, through XBP1s induction as well as RIDD activation, assessed by a decrease in the transcript levels of canonical RIDD targets. A notable finding is the observation that IRE1 and XBP1 accumulated at the ER together with the viral protease/helicase NS3 suggesting that nuclear translocation of XBP1s must be impeded during infection. In line with these findings, we observed that both XBP1 splicing and induction of XBP1s targets genes in ZIKV-infected cells was attenuated when compared to cells treated with tunicamycin, which is a potent inducer of ER stress and the UPR including the IRE1/XBP1s axis. Future studies including a deeper analysis of the induction of transcriptional programs through advanced sequencing techniques will allow to delineate the impact of the IRE1/XBP1s and IRE1/RIDD activation reported here.

Interestingly, we demonstrated that hyperactivation of IRE1 endonuclease activity upon treatment with IXA4 in ZIKV-infected cells resulted in evident changes in the morphology of the ER, which was accompanied with the split of ER-derived structures containing NS3 together with IRE1 and XBP1. Interestingly, RNA FISH and confocal microscopy analyses allowed us to discard the presence of the vRNA in these ER-derived cytoplasmic structures indicating that they might not correspond to VRCs that are split from the ER. Further analyses are required to characterize the identity of these cytoplasmic structures derived from the ER containing NS3, IRE1 and XBP1 observed in infected cells treated with IXA4. However, these analyses also revealed that hyperactivation of IRE1 endonuclease activity results in a significant decrease in ZIKV RNA levels. Whether this phenomenon is due to unspecific cleavage of the vRNA by hyperactivated IRE1 or by the delocalization of a fraction of NS3 from the viral replication sites warrants further investigation.

Our data also suggest a role for IRE1 endonuclease in the control of type-I IFNs during ZIKV replication. Although a previous study showed that pharmacological inhibition of the IRE1 endonuclease activity by treatment with 4μ8C resulted in impaired activation of the IFN-I response triggered by proteosome inhibition in mouse BV-2 microglia [53], our results indicate that inhibition of the endonuclease activity of the UPR sensor by STF-083010 results in increased levels of the IFN-β mRNA in human microglia infected with ZIKV. These discrepancies highlight the versatility of IRE1 in regulating antiviral immunity, which are highly dependent on the species, the cell type, and the stimuli. Indeed, we have recently reported the relevance of a functional IRE1/XBP1 axis for the type-I IFN and pro-inflammatory responses of mouse conventional dendritic cells (cDC1) infected with ZIKV [54], further highlighting species- and cell type-specific contributions of IRE1 to the cellular response towards ZIKV. Indeed, the RIDD branch was required for the generation of small RNA fragments that activated the TANK-binding kinase 1 (TBK1)/interferon regulatory factor 3 (IRF3) pathway in mouse BV-2 microglia [53]. Whether the increase in the IFN-β mRNA levels observed in our human microglia cell line upon inhibition of IRE1 endonuclease activity is due to an impairment of RIDD or whether other branches of the UPR such as PERK or ATF6 are regulating the induction of pro-inflammatory cytokines deserves further investigation. Nevertheless, the interplay between the UPR and the antiviral response may be particularly relevant for ER-replicating viruses such as ZIKV and other members of the flavivirus genus that also represent threads for human health.

Although we acknowledge that the use of a microglia cell line instead of a primary culture and/or microglia derived from iPSCs represents a limitation, our work provides novel insights into the complex interplay between ZIKV infection, the ER stress sensor IRE1 and the antiviral response in humans, highlighting the potential of IRE1 as a target for the development of antivirals to treat ZIKV infections.

## 5. Conclusions

The data presented here demonstrate that a human microglia cell line supports efficient ZIKV replication despite the induction of an antiviral response characterized by the induction of a type-I IFN response together with the pro-inflammatory cytokines IL-6 and IL-8. We also show that ZIKV infection triggers ER stress, activating the IRE1/XBP1s and the IRE1/RIDD branches of the UPR in this human cellular model. We also provide evidence suggesting that IRE1 endonuclease activity is a key regulator of the viral outcome and the control of type-I IFN response in this cell line. These findings highlight the role of IRE1 in the interplay between viral replication and the antiviral host response, providing new insights into the cellular mechanisms underlying ZIKV pathogenesis in the central nervous system.

## Figures and Tables

**Table 1:** Primer sequences used in this study.

Target	Forward (5′-3′)	Reverse (5′-3′)
vRNA	GTCTTGGAACATGGAGG	TTCACCTTGTGTTGGGC
IFN-β	TCTCCTGTTGTGCTTCTCCAC	GCCTCCCATTCAATTGCCAC
IL-6	GCCCAGCTATGAACTCCTTCT	GAAGGCAGCAGGCAACAC
IL-8	GACCACACTGCGCCAACAC	CTTCTCCACAACCCTCTGCAC
XBP1s	AAGAACACGCTTGGGAATGG	CTGCACCTGCTGCGGAC
XBP1u	GACAGAGAGTCAAACTAACGTGG	GTCCAGCAGGCAAGAAGGT
Bloc1s1	CCCAATTTGCCAAGCAGACA	CATCCCCAATTTCCTTGAGTGC
Per1	TATACCCTGGAGGAGCTGGA	AGGAAGGAGACAGCCACTGA
Scara3	CGCTGCCAGAAGAACCTATC	AACCAGAGAGGCCAACACAG
Erjd4	TGGTGGTTCCAGTAGACAAAGG	CTTCGTTGAGTGACAGTCCTGC
Edem1	TTCCCTCCTGGTGGAATTTG	AGGCCACTCTGCTTTCCAAC
18S	TGTGCCGCTAGAGGTGAAATT	TGGCAAATGCTTTCGCTTT

## Data Availability

The original contributions presented in this study are included in the article. Further inquiries can be directed to the corresponding author.

## Abbreviations

The following abbreviations are used in this manuscript:

ATF6	Activating Transcription Factor 6
BiP	Binding immunoglobulin protein
cDC	Conventional dendritic cell
DMSO	Dimethyl Sulfoxide
ER	Endoplasmic Reticulum
FISH	Fluorescent in situ hybridization
IFN-I	Interferon (Type I)
IRF3	Interferon regulatory factor 3
IL	Interleukin (6 or 8)
IRE1	Inositol-requiring enzyme 1
NS3	Nonstructural protein 3
PAMP	Pathogen-Associated Molecular Patterns
PERK	Endoplasmic Reticulum Kinase
PRR	Pattern Recognition Receptors
RIDD	Regulated IRE1α-dependent decay
TBK1	TANK-binding kinase 1
Tn	Tunicamycin
UPR	Unfolded Protein Response
VRC	Viral Replication Compartments
vRNA	Viral RNA
XBP1	X-box Binding Protein 1
ZIKV	Zika virus

## References

[1] Ukoaka B.M., Okesanya O.J., Daniel F.M., Ahmed M.M., Udam N.G., Wagwula P.M., Adigun O.A., Udoh R.A., Peter I.G., Lawal H. (2024). Updated WHO list of emerging pathogens for a potential future pandemic: Implications for public health and global preparedness. Infez. Med..

[2] Plourde A.R., Bloch E.M. (2016). A Literature Review of Zika Virus. Emerg. Infect. Dis..

[3] Wang Y., Ling L., Zhang Z., Marin-Lopez A. (2022). Current Advances in Zika Vaccine Development. Vaccines.

[4] Peng Z.Y., Yang S., Lu H.Z., Wang L.M., Li N., Zhang H.T., Xing S.Y., Du Y.N., Deng S.Q. (2024). A review on Zika vaccine development. Pathog. Dis..

[5] Zhao Z., Li Q., Ashraf U., Yang M., Zhu W., Gu J., Chen Z., Gu C., Si Y., Cao S. (2022). Zika virus causes placental pyroptosis and associated adverse fetal outcomes by activating GSDME. eLife.

[6] Reynoso G.V., Gordon D.N., Kalia A., Aguilar C.C., Malo C.S., Aleshnick M., Dowd K.A., Cherry C.R., Shannon J.P., Vrba S.M. (2023). Zika virus spreads through infection of lymph node-resident macrophages. Cell Rep..

[7] Ferraris P., Cochet M., Hamel R., Gladwyn-Ng I., Alfano C., Diop F., Garcia D., Talignani L., Montero-Menei C.N., Nougairede A. (2019). Zika virus differentially infects human neural progenitor cells according to their state of differentiation and dysregulates neurogenesis through the Notch pathway. Emerg. Microbes Infect..

[8] Veilleux C., Eugenin E.A. (2023). Mechanisms of Zika astrocyte infection and neuronal toxicity. NeuroImmune Pharm. Ther..

[9] Xu P., Shan C., Dunn T.J., Xie X., Xia H., Gao J., Labastida J.A., Zou J., Villarreal P.P., Schlagal C.R. (2020). Role of microglia in the dissemination of Zika virus from mother to fetal brain. PLoS Negl. Trop. Dis..

[10] Stokes C., Whitmore L.S., Moreno D., Malhotra K., Tisoncik-Go J., Tran E., Wren N., Glass I.A., Research L.B.D., Young J.E. (2025). The human neural cell atlas of Zika virus infection in developing brain tissue. Cell Rep. Med..

[11] Tajik S., Farahani A.V., Ardekani O.S., Seyedi S., Tayebi Z., Kami M., Aghaei F., Hosseini T.M., Nia M.M.K., Soheili R. (2024). Zika virus tropism and pathogenesis: Understanding clinical impacts and transmission dynamics. Virol. J..

[12] Jiyarom B., Giannakopoulos S., Strange D.P., Panova N., Gale M., Verma S. (2022). RIG-I and MDA5 are modulated by bone morphogenetic protein (BMP6) and are essential for restricting Zika virus infection in human Sertoli cells. Front. Microbiol..

[13] Plociennikowska A., Frankish J., Moraes T., Del Prete D., Kahnt F., Acuna C., Slezak M., Binder M., Bartenschlager R. (2021). TLR3 activation by Zika virus stimulates inflammatory cytokine production which dampens the antiviral response induced by RIG-I-like receptors. J. Virol..

[14] Schilling M., Bridgeman A., Gray N., Hertzog J., Hublitz P., Kohl A., Rehwinkel J. (2020). RIG-I Plays a Dominant Role in the Induction of Transcriptional Changes in Zika Virus-Infected Cells, which Protect from Virus-Induced Cell Death. Cells.

[15] Shukla A., Rastogi M., Singh S.K. (2021). Zika virus NS1 suppresses the innate immune responses via miR-146a in human microglial cells. Int. J. Biol. Macromol..

[16] Yu Y., Gao C., Wen C., Zou P., Qi X., Cardona C.J., Xing Z. (2022). Intrinsic features of Zika Virus non-structural proteins NS2A and NS4A in the regulation of viral replication. PLoS Negl. Trop. Dis..

[17] Liang Q., Luo Z., Zeng J., Chen W., Foo S.S., Lee S.A., Ge J., Wang S., Goldman S.A., Zlokovic B.V. (2016). Zika Virus NS4A and NS4B Proteins Deregulate Akt-mTOR Signaling in Human Fetal Neural Stem Cells to Inhibit Neurogenesis and Induce Autophagy. Cell Stem Cell.

[18] Ropidi M.I.M., Khazali A.S., Rashid N.N., Yusof R. (2020). Endoplasmic reticulum: A focal point of Zika virus infection. J. Biomed. Sci..

[19] Coyaud E., Ranadheera C., Cheng D., Goncalves J., Dyakov B.J.A., Laurent E.M.N., St-Germain J., Pelletier L., Gingras A.C., Brumell J.H. (2018). Global Interactomics Uncovers Extensive Organellar Targeting by Zika Virus. Mol. Cell Proteom..

[20] Rossignol E.D., Peters K.N., Connor J.H., Bullitt E. (2017). Zika virus induced cellular remodelling. Cell Microbiol..

[21] Long R.K.M., Moriarty K.P., Cardoen B., Gao G., Vogl A.W., Jean F., Hamarneh G., Nabi I.R. (2020). Super resolution microscopy and deep learning identify Zika virus reorganization of the endoplasmic reticulum. Sci. Rep..

[22] Muthuraj P.G., Sahoo P.K., Kraus M., Bruett T., Annamalai A.S., Pattnaik A., Pattnaik A.K., Byrareddy S.N., Natarajan S.K. (2021). Zika virus infection induces endoplasmic reticulum stress and apoptosis in placental trophoblasts. Cell Death Discov..

[23] Tan Z., Zhang W., Sun J., Fu Z., Ke X., Zheng C., Zhang Y., Li P., Liu Y., Hu Q. (2018). ZIKV infection activates the IRE1-XBP1 and ATF6 pathways of unfolded protein response in neural cells. J. Neuroinflamm..

[24] Mufrrih M., Chen B., Chan S.W. (2021). Zika Virus Induces an Atypical Tripartite Unfolded Protein Response with Sustained Sensor and Transient Effector Activation and a Blunted BiP Response. mSphere.

[25] Alfano C., Gladwyn-Ng I., Couderc T., Lecuit M., Nguyen L. (2019). The Unfolded Protein Response: A Key Player in Zika Virus-Associated Congenital Microcephaly. Front. Cell Neurosci..

[26] Ron D., Walter P. (2007). Signal integration in the endoplasmic reticulum unfolded protein response. Nat. Rev. Mol. Cell Biol..

[27] Hetz C., Zhang K., Kaufman R.J. (2020). Mechanisms, regulation and functions of the unfolded protein response. Nat. Rev. Mol. Cell Biol..

[28] Walter P., Ron D. (2011). The unfolded protein response: From stress pathway to homeostatic regulation. Science.

[29] Park S.M., Kang T.I., So J.S. (2021). Roles of XBP1s in Transcriptional Regulation of Target Genes. Biomedicines.

[30] Martinon F., Chen X., Lee A.H., Glimcher L.H. (2010). TLR activation of the transcription factor XBP1 regulates innate immune responses in macrophages. Nat. Immunol..

[31] Smith J.A., Turner M.J., DeLay M.L., Klenk E.I., Sowders D.P., Colbert R.A. (2008). Endoplasmic reticulum stress and the unfolded protein response are linked to synergistic IFN-beta induction via X-box binding protein 1. Eur. J. Immunol..

[32] Cirone M. (2021). ER Stress, UPR Activation and the Inflammatory Response to Viral Infection. Viruses.

[33] Sanchez C.L., Sims S.G., Nowery J.D., Meares G.P. (2019). Endoplasmic reticulum stress differentially modulates the IL-6 family of cytokines in murine astrocytes and macrophages. Sci. Rep..

[34] Garcia-Mesa Y., Jay T.R., Checkley M.A., Luttge B., Dobrowolski C., Valadkhan S., Landreth G.E., Karn J., Alvarez-Carbonell D. (2017). Immortalization of primary microglia: A new platform to study HIV regulation in the central nervous system. J. Neurovirol..

[35] Mutso M., Saul S., Rausalu K., Susova O., Zusinaite E., Mahalingam S., Merits A. (2017). Reverse genetic system, genetically stable reporter viruses and packaged subgenomic replicon based on a Brazilian Zika virus isolate. J. Gen. Virol..

[36] Baz M. (2020). Zika Virus Isolation, Purification, and Titration. Methods Mol. Biol..

[37] Guha P., Kaptan E., Gade P., Kalvakolanu D.V., Ahmed H. (2017). Tunicamycin induced endoplasmic reticulum stress promotes apoptosis of prostate cancer cells by activating mTORC1. Oncotarget.

[38] Grandjean J.M.D., Madhavan A., Cech L., Seguinot B.O., Paxman R.J., Smith E., Scampavia L., Powers E.T., Cooley C.B., Plate L. (2020). Pharmacologic IRE1/XBP1s activation confers targeted ER proteostasis reprogramming. Nat. Chem. Biol..

[39] Papandreou I., Denko N.C., Olson M., Van Melckebeke H., Lust S., Tam A., Solow-Cordero D.E., Bouley D.M., Offner F., Niwa M. (2011). Identification of an Ire1alpha endonuclease specific inhibitor with cytotoxic activity against human multiple myeloma. Blood.

[40] Lum F.M., Low D.K., Fan Y., Tan J.J., Lee B., Chan J.K., Renia L., Ginhoux F., Ng L.F. (2017). Zika Virus Infects Human Fetal Brain Microglia and Induces Inflammation. Clin. Infect. Dis..

[41] Li Y., Shi S., Xia F., Shan C., Ha Y., Zou J., Adam A., Zhang M., Wang T., Liu H. (2021). Zika virus induces neuronal and vascular degeneration in developing mouse retina. Acta Neuropathol. Commun..

[42] Kolpikova E.P., Tronco A.R., Hartigh A.B.D., Jackson K.J., Iwawaki T., Fink S.L. (2020). IRE1alpha Promotes Zika Virus Infection via XBP1. Viruses.

[43] Huang Y., Lin Q., Huo Z., Chen C., Zhou S., Ma X., Gao H., Lin Y., Li X., He J. (2020). Inositol-Requiring Enzyme 1alpha Promotes Zika Virus Infection through Regulation of Stearoyl Coenzyme A Desaturase 1-Mediated Lipid Metabolism. J. Virol..

[44] Kaufman R.J., Cao S. (2010). Inositol-requiring 1/X-box-binding protein 1 is a regulatory hub that links endoplasmic reticulum homeostasis with innate immunity and metabolism. EMBO Mol. Med..

[45] Fu F., Doroudgar S. (2022). IRE1/XBP1 and endoplasmic reticulum signaling—from basic to translational research for cardiovascular disease. Curr Opin Physiol.

[46] Zeng L., Liu Y.P., Sha H., Chen H., Qi L., Smith J.A. (2010). XBP-1 couples endoplasmic reticulum stress to augmented IFN-beta induction via a cis-acting enhancer in macrophages. J. Immunol..

[47] Chen Z., Zhong D., Li G. (2019). The role of microglia in viral encephalitis: A review. J. Neuroinflamm..

[48] Martinez Viedma M.D.P., Pickett B.E. (2018). Characterizing the Different Effects of Zika Virus Infection in Placenta and Microglia Cells. Viruses.

[49] Borst K., Dumas A.A., Prinz M. (2021). Microglia: Immune; non-immune functions. Immunity.

[50] Oliveira F.B.C., Freire V., Coelho S.V.A., Meuren L.M., Palmeira J.D.F., Cardoso A.L., Neves F.A.R., Ribeiro B.M., Arganaraz G.A., Arruda L.B. (2023). ZIKV Strains Elicit Different Inflammatory and Anti-Viral Responses in Microglia Cells. Viruses.

[51] Manet C., Mansuroglu Z., Conquet L., Bortolin V., Comptdaer T., Segrt H., Bourdon M., Menidjel R., Stadler N., Tian G. (2022). Zika virus infection of mature neurons from immunocompetent mice generates a disease-associated microglia and a tauopathy-like phenotype in link with a delayed interferon beta response. J. Neuroinflamm..

[52] Gorman M.J., Caine E.A., Zaitsev K., Begley M.C., Weger-Lucarelli J., Uccellini M.B., Tripathi S., Morrison J., Yount B.L., Dinnon K.H. (2018). An Immunocompetent Mouse Model of Zika Virus Infection. Cell Host Microbe.

[53] Studencka-Turski M., Cetin G., Junker H., Ebstein F., Kruger E. (2019). Molecular Insight Into the IRE1alpha-Mediated Type I Interferon Response Induced by Proteasome Impairment in Myeloid Cells of the Brain. Front. Immunol..

[54] Guzman-Rodriguez M., Hernandez-Diaz T., Lisboa P., Lopez-Schettini J., Sanhueza S., Leyton L., Iwawaki T., Soto-Rifo R., Osorio F. (2025). The IRE1/XBP1s Axis Regulates Innate Immune Responses in Conventional Dendritic Cells During ZIKV Infection. FASEB J..

